# An Automated Literature Review Tool (LiteRev) for Streamlining and Accelerating Research Using Natural Language Processing and Machine Learning: Descriptive Performance Evaluation Study

**DOI:** 10.2196/39736

**Published:** 2023-09-15

**Authors:** Erol Orel, Iza Ciglenecki, Amaury Thiabaud, Alexander Temerev, Alexandra Calmy, Olivia Keiser, Aziza Merzouki

**Affiliations:** 1 Institute of Global Health University of Geneva Geneva Switzerland; 2 Médecins Sans Frontières Geneva Switzerland; 3 HIV/AIDS Unit Division of Infectious Diseases Geneva University Hospital Geneva Switzerland

**Keywords:** LiteRev, literature review, natural language processing, machine learning, automation, clustering, topic, acute, early, HIV

## Abstract

**Background:**

Literature reviews (LRs) identify, evaluate, and synthesize relevant papers to a particular research question to advance understanding and support decision-making. However, LRs, especially traditional systematic reviews, are slow, resource-intensive, and become outdated quickly.

**Objective:**

LiteRev is an advanced and enhanced version of an existing automation tool designed to assist researchers in conducting LRs through the implementation of cutting-edge technologies such as natural language processing and machine learning techniques. In this paper, we present a comprehensive explanation of LiteRev’s capabilities, its methodology, and an evaluation of its accuracy and efficiency to a manual LR, highlighting the benefits of using LiteRev.

**Methods:**

Based on the user’s query, LiteRev performs an automated search on a wide range of open-access databases and retrieves relevant metadata on the resulting papers, including abstracts or full texts when available. These abstracts (or full texts) are text processed and represented as a term frequency-inverse document frequency matrix. Using dimensionality reduction (pairwise controlled manifold approximation) and clustering (hierarchical density-based spatial clustering of applications with noise) techniques, the corpus is divided into different topics described by a list of the most important keywords. The user can then select one or several topics of interest, enter additional keywords to refine its search, or provide key papers to the research question. Based on these inputs, LiteRev performs a k-nearest neighbor (k-NN) search and suggests a list of potentially interesting papers. By tagging the relevant ones, the user triggers new k-NN searches until no additional paper is suggested for screening. To assess the performance of LiteRev, we ran it in parallel to a manual LR on the burden and care for acute and early HIV infection in sub-Saharan Africa. We assessed the performance of LiteRev using true and false predictive values, recall, and work saved over sampling.

**Results:**

LiteRev extracted, processed, and transformed text into a term frequency-inverse document frequency matrix of 631 unique papers from PubMed. The topic modeling module identified 16 topics and highlighted 2 topics of interest to the research question. Based on 18 key papers, the k-NNs module suggested 193 papers for screening out of 613 papers in total (31.5% of the whole corpus) and correctly identified 64 relevant papers out of the 87 papers found by the manual abstract screening (recall rate of 73.6%). Compared to the manual full text screening, LiteRev identified 42 relevant papers out of the 48 papers found manually (recall rate of 87.5%). This represents a total work saved over sampling of 56%.

**Conclusions:**

We presented the features and functionalities of LiteRev, an automation tool that uses natural language processing and machine learning methods to streamline and accelerate LRs and support researchers in getting quick and in-depth overviews on any topic of interest.

## Introduction

Recently, the traditional emphasis of literature reviews (LRs) in identifying, evaluating, and synthesizing all relevant papers to a particular research question has shifted toward mapping research activity and consolidating existing knowledge [1]. Despite this broader scope, manual LRs are still error-prone, time- and resource-intensive, and have become ever more challenging over the years due to the increasing number of papers published in academic databases. It is estimated that within 2 years of publication, about one-fourth of all LRs are outdated, as reviewers fail to incorporate new papers on their topic of interest [2,3].

To shorten the time to completion, automation tools have been developed to either fully automate or semiautomate one or more specific tasks involved in conducting an LR, such as screening titles and abstracts [4,5], sourcing full texts, or automating data extraction [6]. In addition, recent advances in natural language processing (NLP) and machine learning (ML) have produced new techniques that can accurately mimic manual LRs faster and at lower costs [7-9]. In Vienna, in 2015, the International Collaboration for the Automation of Systematic Reviews was initiated to establish a set of principles to enable tools to be developed and integrated into toolkits [10].

In 2020, our group of researchers started developing an automation tool for LRs [11] in order to obtain a comprehensive overview of the sociobehavioral factors influencing HIV prevalence and incidence in Malawi. In this paper, we propose an updated version of the tool called LiteRev, which overcomes some of the shortcomings of the previous version. While previously restricted to Paperity, PubMed, PubMed Central, JSTOR, and arXiv, the search now includes 2 additional primary preprint services in the field of epidemiology and medical sciences, bioRxiv and medRxiv, and CORE, a large collection of open-access research papers. In addition, in our previous tool, the search was systematically performed on the papers’ full texts, and references were included in the processed text. In LiteRev, the user can choose to focus on the abstract or on the full text and include or exclude the references. In addition, multiple parallel application programming interface (API) connections to each database have been implemented, allowing for faster retrieval of papers. In the last years, NLP and ML have rapidly evolved, and LiteRev makes use of the most recent text processing, embedding, and clustering techniques. Finally, we added a k-nearest neighbor (k-NN) search module that allows the user to find papers of high similarities with key papers to the research question.

To assess the performance of LiteRev, we conducted a manual LR on the burden and care for acute and early HIV infection (AEHI) in sub-Saharan Africa using one open-access database, PubMed, and 2 subscription-based databases, Embase and Web of Science. AEHI contributes to continuous HIV transmission despite global achievements in HIV control [12,13]. Acute HIV infection is a brief period between viral acquisition and appearance of HIV antibodies, characterized by extremely high viral load values, seeding of viral reservoirs, and disproportionally high likelihood of onward transmission [14-16]. Diagnosing acute HIV infection is challenging: the symptoms are often unspecific, the infection cannot be detected with antibody-detecting rapid diagnostic tests, and the tests detecting antigens or viruses are more complex and expensive. Nevertheless, the testing and care for AEHI have been part of guidance and practice of routine HIV care in high-income countries for many years [17-19]; yet in sub-Saharan Africa, the diagnosis and care for AEHI are almost nonexistent, and current WHO testing guidelines provide no guidance [20,21]. The objective of the proposed LR is to summarize the current knowledge on the burden of AEHI in sub-Saharan Africa and existing models of care to inform future public health interventions. All papers available by December 20, 2022, and related to burden and care for AEHI in sub-Saharan Africa were retrieved, and after removing duplicates, unique papers were screened for relevance. After screening, papers from PubMed identified as relevant by the manual LR were compared to the list of suggested papers by LiteRev. We discussed the performance using standard classification metrics such as true and false predictive values, recall, and work saved over sampling (WSS).

## Methods

### LiteRev

#### Metadata Collection and Text Processing

Based on the user’s query, LiteRev performs an automated search, using the corresponding APIs, on 8 different open-access databases: PubMed, PubMed Central, CORE, JSTOR, Paperity, arXiv, bioRxiv, and medRxiv. Available metadata, that is, list of authors and their affiliations, MeSH keywords, digital object identifier, title, abstract, publication date, journal provider, and URL of the PDF version of the full text paper, are retrieved and stored in a PostgreSQL database hosted on the local machine of the user. If the full text is not available as metadata, it is extracted automatically from the available PDF file, then, references, acknowledgments, and other unnecessary terms are removed, and the remaining text is checked to confirm that it still satisfies the search terms. To identify duplicate papers, the tool compares the title and abstract of papers. If duplicates are identified, the metadata of the papers are compared to check for any discrepancies. In cases where there are discrepancies, information is merged from different sources to collect as much information as possible on the same paper. Depending on the user’s needs and requirements, LiteRev can be performed on the abstract or on the full text.

NLP has evolved rapidly, and, in particular, some powerful tools were developed to process text data much more efficiently. We included those features in LiteRev (Gensim [22] and spaCy [23]). After removing papers with empty text, emails, newline characters, single quotes, internet addresses, and punctuation are stripped, and papers that do not fulfill the languages (one or multiple) chosen by the user are discarded. Sentences are then split into words and lemmatized to remove as many variations of the same word as possible. Words belonging to a list of stop words (ie, words that are not informative) and words with less than 3 characters are also removed. Next, bigrams, trigrams, and four-grams (ie, the combination of 2, 3, and 4 words) are created using a probabilistic measure. In practice, n-gram models are highly effective in modeling language data. Finally, we remove words that are in only 1 paper or words that occur too often (ie, in more than 60% of the corpus) to have a significant meaning.

#### Clustering and Topic Modeling

Topic modeling allows organizing documents into clusters based on similarity and identifying abstract topics covered by similar papers. In LiteRev, it allows the user to broaden the search strategy and get a more comprehensive and organized overview of the corpus. It can also help to quickly discard a pool of papers when searching the literature for a specific topic and significantly reduce the amount of text to verify manually.

After abstracts or full texts are processed, each paper’s remaining words (namely, bags of words) are represented as a term frequency-inverse document frequency (TF-IDF) matrix, which is computed using the *Scikit-Learn* package [24]. A TF-IDF matrix is similar to a document (in row) and word (in column) co-occurrence matrix normalized by the number of papers in which the word is present. Less meaningful words, often present in the corpus, get a lower score. Because of the often-high dimension of the TF-IDF matrix (size of corpus × size of vocabulary), it is needed to embed the matrix using a pairwise controlled manifold approximation (PaCMAP) dimensionality reduction technique [25]. The corpus is then divided into different clusters using the hierarchical density-based spatial clustering of applications with noise (HDBSCAN) algorithm [26].

PaCMAP and HDBSCAN have several important hyperparameters that need to be determined. Table S1 in Multimedia Appendix 1 represents the 4 hyperparameters involved and the ranges of their possible values. To find the best set of hyperparameters possible, we use the Tree-structured Parzen Estimator algorithm implemented by the *Optuna* package [27] and store the results of 500 trials in the previously created PostgreSQL database. The density-based clustering validation (DBCV), a weighted sum of “validity index” values of clusters [28], is the considered performance metric to compare the different sets. Its value varies between 0 and 1 when used with HDBSCAN, with larger values providing better clustering solutions. This metric takes the noise into account and captures the shape property of clusters via densities and not distances. For coherency check, another metric is computed, the Silhouette coefficient, which measures cluster cohesiveness and separation with an index between –1 and 1, with larger values providing better clustering solutions [29].

If after 500 trials, the DBCV score is below 0.5, another round of 500 trials is performed, and so on until a DBCV score equal to or above 0.5 is reached. Once the values of the hyperparameters that maximize the DBCV score are determined, obtained clusters that are larger than 25% of the corpus are clustered again with the same entire procedure described above (starting from the text processing). Once each cluster is smaller than 25% of the corpus, its 10 most important words are extracted using the *YAKE* package [30] to ensure interpretability and define topics. This supports the user in getting a quick overview of the corpus and, if desired, to select one or more topics of interest for further exploration. They can then also enter additional keywords to refine this search.

#### Nearest Neighbors

LiteRev allows the user to define or add papers in the corpus that are considered as being key to the research question. The key papers for the case study were proposed by one of the coauthors (IC), who is working on the topic of acute HIV infection. The papers were chosen by the coauthor from the previously identified literature based on a nonsystematic search and from the references of key review papers if they fulfilled inclusion criteria (see Manual LR in the Methods section). Using the k-NN algorithm from the *Scikit-Learn* package [24], a list of potentially relevant papers is provided to the user. Papers deemed to be relevant are tagged by the user and considered as new key papers. This process is iterated as long as relevant papers are being identified (generally 3 to 4 iterations). The initial value of the hyperparameter k, which represents the number of nearest neighbors to be selected, is equal to the value of the number of neighbors for PaCMAP obtained at the first clustering process. The dimension space is the same as the number of dimensions obtained during the embedding process by PaCMAP.

**Figure 1:**
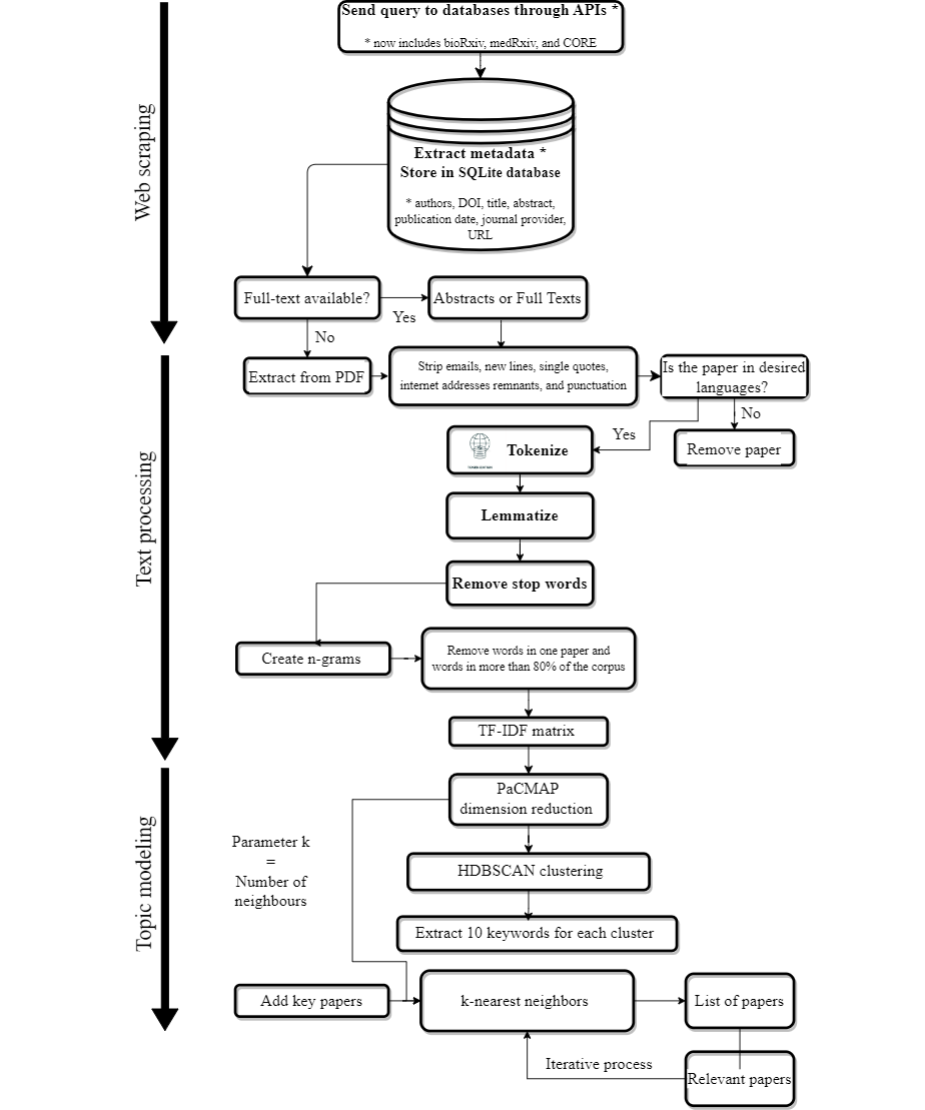
Diagram of LiteRev process. API: application programming interface; DOI: digital object identifier; HDBSCAN: hierarchical density-based spatial clustering of applications with noise; PaCMAP: pairwise controlled manifold approximation; TF-IDF: term frequency-inverse document frequency.

The list of relevant papers from the k-NN search or a list of papers about one or more topics can then be exported in a CSV or HTML format, and their PDF retrieved and stored in a zip folder. For visualization and further exploration, a web-based 2D representation of the corpus is available in an HTML format. Every dot, colored according to the cluster it belongs to, represents a paper with the following available information: date, title, 10 most important keywords of the cluster’s topic, and the cluster number. When clicking on a paper (dot), direct access to the full text is provided using the URL. Figure 1 shows the entire process flow of LiteRev.

### Manual LR

The manual LR aimed at summarizing the current evidence on the burden and care provided for AEHI in sub-Saharan Africa, to inform policy, practice, and research in the future, to address the following questions: What is the prevalence of AEHI in sub-Saharan Africa among people being tested for HIV? What models of care have been used for AEHI diagnosis and care, including treatment, partner notifications, and behavior change? What linkage to care has been reached? and What facilitators and barriers to AEHI care were identified?

We searched all papers in PubMed, Embase, and Web of Science related to burden and care for AEHI in sub-Saharan Africa that were published from the inception of the databases to December 20, 2022, using the query: “(”early hiv“ OR ”primary hiv“ OR ”acute hiv“ OR ”HIV Human immuno deficiency virus“ OR (”Window period“ AND HIV)) AND (”Africa South of the Sahara“ OR ”Central Africa“ OR ”Eastern Africa“ OR ”Southern Africa“ OR ”Western Africa“ OR ”sub-saharan africa“ OR ”subsaharan africa“ OR angola OR benini OR botswana OR ”burkina faso“ OR burundi OR cameroon OR ”cape verde“ OR ”central africa“ OR ”central african republic“ OR chad OR comoros OR congo OR ”cote d ivoire“ OR ”democratic republic congo“ OR djibouti OR ”equatorial guinea“ OR eritrea OR eswatini OR ethiopia OR gabon OR gambia OR ghana OR guinea OR ”guinea-bissau“ OR kenya OR lesotho OR liberia OR madagascar OR malawi OR mali OR mayotte OR mozambique OR namibia OR niger OR nigeria OR rwanda OR sahel OR ”sao tome and principe“ OR senegal OR ”sierra leone“ OR somalia OR ”south africa“ OR ”south sudan“ OR sudan OR tanzania OR togo OR uganda OR zambia OR zimbabwe)”. This query is specific to PubMed syntax and is the exact same for both the manual LR and LiteRev. Syntax-specific queries for the manual LR in Embase and Web of Science are shown in Multimedia Appendix 1. Papers retrieved from Embase and Web of Science have not been used by LiteRev and will not be part of the comparison and performance assessment, but their results will be discussed in the Results and Discussion sections.

The studies were included if they described AEHI prevalence among the population tested for HIV or describe the diagnostic strategy, model of care or linkage to care for AEHI, including studies looking at perceptions and barriers among patients and staff. Only studies conducted in sub-Saharan Africa were included. We followed the Joanna Briggs Institute methodology for conducting LRs [31], and papers identified by the databases were uploaded into Rayyan (Rayyan) [32]. Duplicates were deleted, and the screening process, on titles and abstracts, was conducted independently by 2 reviewers (EO and IC). Selected papers were further manually screened based on full text for eligibility against inclusion criteria. LiteRev was run in parallel on the abstracts only, but results were compared both to the title or abstract screening phase and the full-text screening phase of the manual LR.

### Performance Comparison

To assess the performance of LiteRev, we compared the results from the manual LR to the same review conducted using LiteRev. Relevant and not relevant papers, as identified by the manual LR during the title or abstract screening phase and the full-text screening phase, were defined as true labels. Suggested and not suggested papers by LiteRev were considered as predicted labels. Based on these figures, 2 confusion matrices were produced. Positive and negative predictive values (% of relevant and not relevant papers correctly identified; PPV and NPV), recall (number of relevant papers identified using LiteRev among those identified using manual review), and WSS [33,34] were computed and discussed.

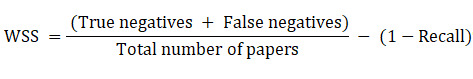



where true negatives are the number of nonrelevant abstracts that were correctly identified as nonrelevant by LiteRev, that is, that were not suggested by LiteRev for screening, and false negatives are the number of relevant abstracts incorrectly classified as nonrelevant by LiteRev.

### Ethical Considerations

No ethics approval was applied as the underlying data are not subject to any approval. The data are publicly available metadata from scientific papers.

## Results

### Text Processing and Topic Modeling

Based on the search strategy described in the Methods section, we obtained 653 papers with metadata directly from PubMed and added 1 key paper given by the user that was not present in the list of retrieved papers. After removing duplicates (n=3), papers with no abstract available (n=15), those not in English (n=3), and empty abstract after text processing (n=2), 631 unique papers were transformed in a TF-IDF matrix comprised of 631 rows representing the corpus and 3136 columns representing the unique words (vocabulary), including n-grams.

For the first embedding and clustering process, a DBCV score of 0.533 was obtained after the first 500 trials with the following best set of hyperparameters: PaCMAP: 310 dimensions and 18 neighbors; and HDBSCAN: minimum cluster size of 30 and minimum samples of 7. This resulted in 5 main clusters composed of, respectively, 203, 193, 169, 35, and 31 papers. The 3 largest main clusters contained more than 25% of the total number of papers in the corpus, which triggered 3 additional text processing, embedding, and clustering processes. The best set of hyperparameters for these additional processes can be found in Table S1 in Multimedia Appendix 1.

**Figure 2:**
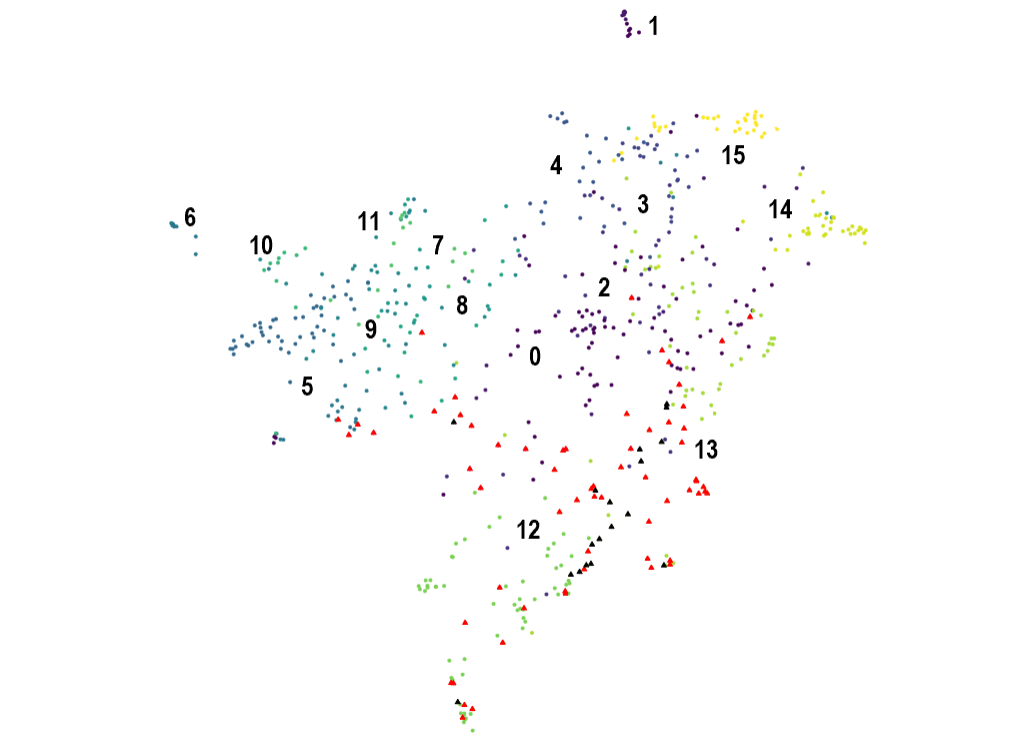
2D representation of the corpus with the 16 clusters. Black triangles represent the 18 key papers and red triangles represent the 64 relevant papers correctly identified by LiteRev.

In the end, the pool of 203 papers was split into 5 clusters (with, respectively, 98, 41, 25, 21, and 18 papers), the pool of 193 papers into 7 clusters (with, respectively, 47, 40, 37, 22, 20, 14, and 13 papers), and the pool of 169 papers into 2 clusters (with, respectively, 87 and 82 papers). In total, the corpus of 631 papers was divided into 16 clusters ranging from 13 to 98 papers. Figure 2 shows the 2D map of the corpus with the 16 clusters identified. Table 1 shows the corresponding 16 topics grouped by main topics described by their 10 most important keywords and the number of papers in each.

**Table 1:** The 16 topics grouped by 5 main topics (in bold) with the 10 most important keywords, the number of papers, and the number of relevant papers in total (key papers).

Topic			Keywords		Papers, n		Relevant papers (key papers), n (%)
**Woman, patient, risk, year, treatment, associate, month, incidence, testing, care**							
	0	Woman, risk, year, incidence, high, man, partner, transmission, sexual, testing		98		9 (1)	
	1	Cart, month, initiation, group, treatment, viral, rna, child, infant, week		18		0 (0)	
	2	Risk, high, health, day, score, aehi, prevalence, care, diagnosis, population		41		6 (2)	
	3	Patient, treatment, care, late, diagnosis, associate, testing, aor, datum, initiation		21		0 (0)	
	4	Patient, disease, adult, infect, lymphadenopathy, cell, tuberculous, lymphadenitis, associate, present		25		0 (0)	
**Cell, viral, response, virus, subtype, individual, primary, isolate, antibody, infect**							
	5	Antibody, response, neutralize, vaccine, isolate, neutralization, epitope, env, primary, individual		47		0 (0)	
	6	Subtype, resistance, drug, sequence, mutation, diversity, strain, primary, patient, recombinant		40		3 (0)	
	7	Response, specific, associate, increase, immune, ifn, early, gag, point, level		37		0 (0)	
	8	Level, viremia, acute, associate, early, individual, infect, load, cytokine, set		20		1 (0)	
	9	Load, early, copy, log, plasma, subtype, woman, time, african, rna		22		5 (1)	
	10	Isolate, primary, tropic, individual, derive, clone, strain, infect, dual, sequence		13		0 (0)	
	11	Response, immune, phi, specific, control, activation, plasma, individual, acute, cytokine		14		0 (0)	
**Test, testing, blood, ahi, risk, acute, positive, care, sample, assay**							
	12	Blood, assay, sample, donor, positive, risk, incidence, antibody, estimate, acute		82		21 (7)	
	13	Ahi, care, participant, health, intervention, patient, diagnosis, early, acute, risk		87		37 (7)	
**Infant, mother, week, transmission, child, month, age, woman, test, infect**							
	14	Infant, mother, week, transmission, child, month, age, woman, test, infect		35		0 (0)	
**Child, year, mortality, age, infect, treatment, patient, associate, month, clinical**							
	15	Child, year, mortality, age, infect, treatment, patient, associate, month, clinical		31		0 (0)	

### Manual LR

Using the search query described in the Methods section, 1721 records were retrieved, among which 653 were from PubMed and 1067 records from 2 subscription-based databases, namely, Embase and Web of Science. In total, 879 records were excluded after removing duplicates, empty abstracts, and papers that were not written in English. This resulted in 631 unique papers in PubMed and 211 unique papers in Embase and Web of Science. We also removed the 18 key papers from the PubMed corpus before the screening phases. In total, 613 papers in PubMed were screened at the title and abstract level, and 87 of them were relevant to the research question. After the full-text screening phase on these 87 relevant papers, we found 48 papers to be relevant to the manual LR.

**Figure 3:**
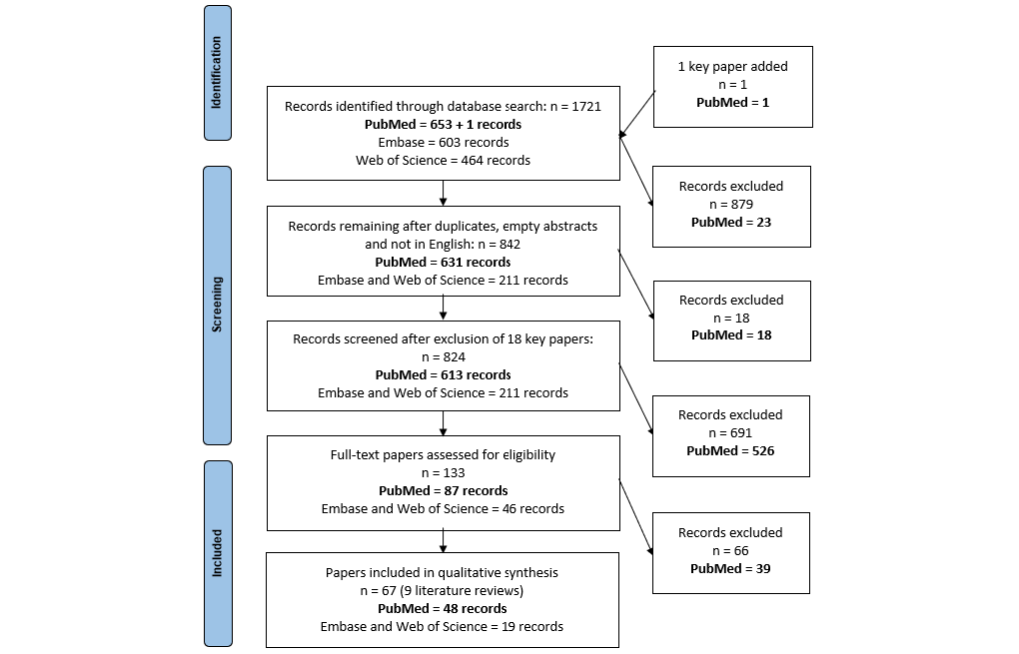
PRISMA (Preferred Reporting Items for Systematic Reviews and Meta-Analyses) diagram of the manual literature review related to burden and care for acute and early HIV infection in sub-Saharan Africa.

Out of the 211 unique papers from Embase and Web of Science, 46 papers were found relevant to the research question after the title or abstract screening phase (ie, 34.6% of the 133 relevant papers), and 19 after the full-text screening phase (ie, 28.4% of the 67 relevant papers; Figure 3). From these 19 relevant papers, 3 were conference abstracts, and 1 paper was kept only based on its title and abstract as the full text could not be found. These 221 papers were not part of PubMed, and hence, not available to LiteRev.

### Nearest Neighbors Search and Performance Comparison

One coauthor (IC) provided a list of 18 key papers. With these 18 key papers, we performed a k-NN search on the corpus, embedded into 310 dimensions, with k=18, the number of the nearest neighbors for PaCMAP that maximized the DBCV score of the first clustering process. The first k-NN search suggested 110 papers, including 45 of the relevant papers identified by the manual LR title or abstract screening (precision of 41%). Based on these 45 relevant papers, the second k-NN iteration suggested 26 additional papers out of which 8 were confirmed as relevant (precision of 31%). The third iteration found 9 more relevant papers out of 38 papers suggested (precision of 24%). The fourth and last iteration suggested 19 papers out of which 1 was relevant (precision of 5%).

In total, 193 papers out of the 613 papers were suggested by LiteRev. Suggested papers included 64 of the 87 papers identified as relevant during the title or abstract screening of the manual LR. Figure 3 maps the key papers (black triangles) and the relevant papers (red triangles) identified at the title or abstract screening level of the manual LR and that were correctly classified as relevant by LiteRev. Table 1 indicates the number of key papers and the number of relevant papers on each topic.

**Figure 4:**
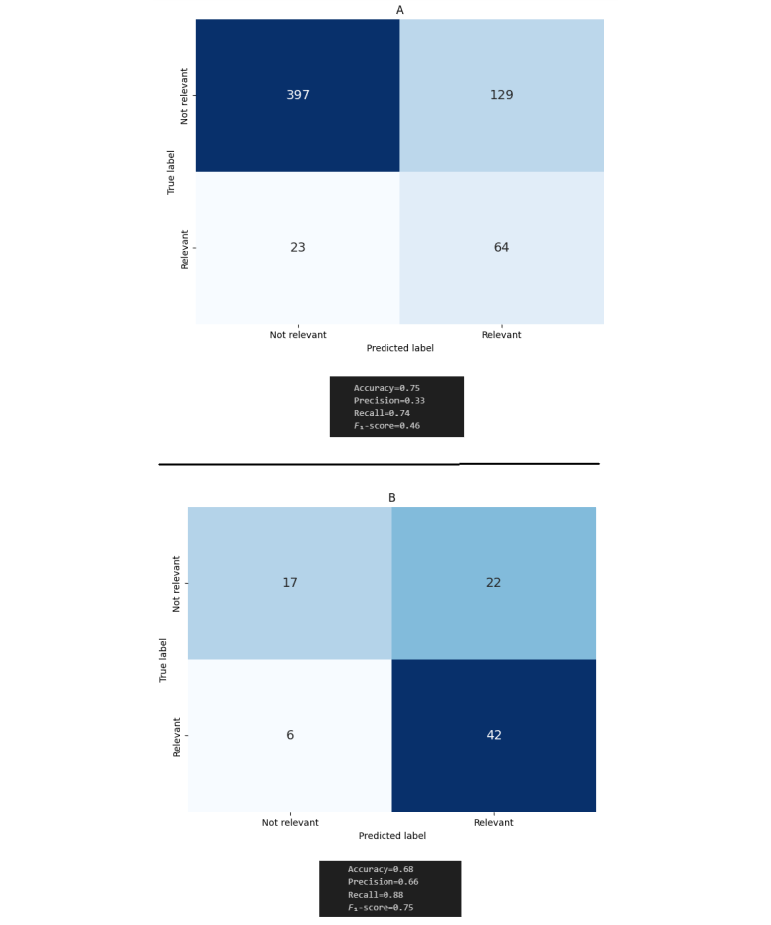
Confusion matrices based on the results of (top panel) the title or abstract screening and (bottom panel) full-text screening performed during the manual literature review.

Figure 4 (top panel) summarizes the above results and represents the confusion matrix between LiteRev (predicted labels) and the manual LR (true labels) after the title or abstract screening phase. Based on these numbers, the PPV was 33.2%, the NPV was 94.5%, and the recall was 73.6%, which led to a WSS of 42.1%.

The 64 relevant papers found by LiteRev belonged essentially to 2 topics (30 relevant papers in one and 14 relevant papers in the other). The topic that contained 30 relevant papers had 87 papers in total and covered early diagnosis, care seeking, and interventions during the acute HIV infection stage (keywords: ahi, care, participant, health, intervention, patient, diagnosis, early, acute, risk). The topic that contained 14 relevant papers had 82 papers and covered the detection of AEHI by antibody assays and incidence estimate (keywords: blood, assay, sample, donor, positive, risk, incidence, antibody, estimate, acute). Screening 53 additional papers (those not suggested by the k-NN search) from these 2 topics would allow the user to identify 3 additional relevant papers.

After the full-text screening phase of the manual LR, 48 out of the 87 relevant papers from the title and abstract screening phase were deemed relevant to the research question. The 64 papers suggested by LiteRev (based on abstracts only) included 42 out of the 48 papers confirmed as relevant after the full-text screening phase of the manual LR. Figure 4 (bottom panel) summarizes the above results and represents the confusion matrix between LiteRev (predicted labels) and the manual LR (true labels) after the full-text screening phase. Based on these numbers, the PPV was 65.6%, the NPV was 26.1%, and the recall was 87.5%, which led to an additional WSS of 13.9% for an overall WSS of 56% compared to the manual LR.

### Processing Time

The processing time represents the overall computation time taken by LiteRev to complete the entire process of metadata retrieval, processing, clustering, and neighbor search. It does not include the time that the user took to check the relevance of the suggested papers. The percentage of time saved by the user is expressed by the WSS metric.

It took 5 minutes for LiteRev to retrieve the metadata of the 653 papers and text process the remaining 631 abstracts and transform it into a TF-IDF matrix. Each trial of the optimization process with a specific set of hyperparameters required on average 1 minute of computation. With 3000 trials in total (500 for the main clustering process, 1000 for the first 2 additional clustering processes, and 500 for the last one) run sequentially, this led to an additional 50 hours, that is, roughly 2 days, to complete the entire optimization process. This computation time can be substantially reduced by running the trials in parallel. Finally, the nearest neighbors are obtained almost instantaneously.

## Discussion

### Principal Results

We presented LiteRev, an automation tool that uses NLP and ML methods to support researchers in different steps of a manual LR. The identification of papers to be included in an LR is a critical and time-intensive process, with the majority of time spent in screening thousands of papers for relevance. By combining text processing, literature mapping, topic modeling, and similarity-based search, LiteRev provides a fast and efficient way to remove duplicates, select papers from specific languages, visualize the corpus on a 2D map, identify the different topics covered when addressing the research question, and suggest a list of potentially relevant papers to the user based on their input (eg, prior knowledge of key papers).

Preliminary usage of LiteRev showed that it significantly reduced the researcher’s workload and overall time required to perform an LR. Compared to a manual LR, LiteRev correctly identified 87.5% of the 48 relevant papers (recall), by screening only 31.5% (193/631 papers) of the whole corpus, which corresponds to a total WSS of 56% at the end of the full-text screening phase. In addition, the actual time spent on running LiteRev and retrieving the results was relatively short, and the user was free in the meantime to focus on other work. The text processing and the nearest neighbors search took no more than 5 minutes of computation for 631 papers.

With its topic modeling capability, LiteRev aims at summarizing current evidence on a specific research question to inform policy, practice, and research. For our use case, LiteRev identified 5 main topics and 16 different topics related to AEHI in sub-Saharan Africa, allowing the researcher to have an overview of the different perspectives related to this research question. Finding 61 out of the 105 relevant papers after the title and abstract screening phase (including the key papers) in only 2 topics validates the quality of the clustering.

### Limitations

LiteRev is currently limited to open-access databases that provide free APIs to abstract or full-text papers. Databases often used for LRs, such as Embase or Web of Science do not provide API access, require a subscription for accessing full-text papers, or do not allow for text mining and ML analysis. Hence, 19 relevant papers identified in Embase or Web of Science were not available to LiteRev. In addition, when performed on full texts, LiteRev currently works on digitally generated PDFs but not on image-only (scanned) PDFs.

Another limitation concerns the possibility of sharing the list of potentially relevant papers with other users or reviewers. LiteRev does not offer this functionality yet; hence, double screening of papers and comparison of results are not possible at the moment. To overcome this limitation, the user has the option to export their list of papers into a CSV format, which can be uploaded on Rayyan or other similar software for systematic reviews.

As of today, LiteRev is still intended to complement rather than replace full systematic reviews. Finally, by January 2023, no public web-based user interface is available yet.

### Comparisons and Future Work

The systematic review tool [35] maintains a searchable database of tools that can be used to assist in many aspects of LR studies, several of which aim to semiautomate parts of the review process. At the end of February 2022, we identified 14 tools (out of which 9 were free) designed to semiautomate searching and screening with only 4 of them providing text analysis functionalities (scite.ai, SRDB.PRO, StArt, and Sysrev). In addition, since the beginning of 2022, a collaborative team at Utrecht University created a repository that aims to give an overview and comparison of software used for systematically screening large amounts of textual data using ML [36]. The process of the initial selection of the software tools is described in the Open Science Framework [37]. Out of the 9 software listed, 4 were free and 2 were in addition open-source (ASReview [38] and FASTREAD [39]). Most of them were using TF-IDF for feature extractions with other methods being Word(Doc)2Vec, and one also using Sentence Embeddings Using Siamese BERT Networks (ASReview). All of them were then using classifiers (mainly support vector machine) with or without balancing techniques with ASReview allowing users to choose between different algorithms. None were using a combination of unsupervised learning techniques (PaCMAP and HDBSCAN) in conjunction with a k-NN search. When we have fulfilled the inclusion criteria, we plan to make a pull request and add LiteRev to the overview.

LiteRev is developed in an iterative way with continuous integration of feedbacks from users, and its modules can easily be updated or replaced depending on the needs of the users and the technical evolutions. We are further developing LiteRev by proposing a web application with a user-friendly interface and by adding more functionality in order to better automate the different stages of an LR. We are also planning to implement a living review [40] by retrieving new papers on each research question in our database (eg, “HIV” AND “Africa”) on a regular basis (eg, every month), and each new paper will be text processed and assigned to the topic it belongs to using a predictive algorithm. Although we compared the performance of LiteRev with 1 manual LR in this paper, we plan to perform additional similar comparisons and performance evaluations in the future using other published LRs covering different topics.

### Conclusions

We presented LiteRev, an automation tool that uses NLP and ML techniques to support, facilitate, and accelerate the conduction of LRs providing aid and automation to different steps involved in this process. Its different modules (retrieval of papers’ metadata from open-access databases using a search query, processing of texts, embedding and clustering, and finding of nearest neighbors) can easily be updated or replaced depending on the needs of the users and the technical evolutions. As more papers are published every year, LiteRev not only has the potential to simplify and accelerate LRs, but it also has the capability of helping the researcher get a quick and in-depth overview of any topic of interest.

## Appendix

Multimedia Appendix 1 Query for Embase and Web of Science, along with the hyperparameters definition, range and values for each clustering.

## Abbreviations

AEHI: acute and early HIV infection
API: application programming interface
DBCV: density-based clustering validation
HDBSCAN: hierarchical density-based spatial clustering of applications with noise
k-NN: k-nearest neighbor
LR: literature review
ML: machine learning
NLP: natural language processing
NPV: negative predictive value
PaCMAP: pairwise controlled manifold approximation
PPV: positive predictive value
TF-IDF: term frequency-inverse document frequency
WSS: work saved over sampling

## Data Availability

The data sets generated and analyzed during this study are available on the Open Science Framework platform named “Burden and care for acute and early HIV infection in sub-Saharan Africa: a scoping review protocol” [41]. A CSV file with the metadata of the 654 papers before text processing and the term frequency-inverse document frequency matrix (and term frequency-inverse document frequency vectorizer) in a pickle format have been uploaded and are freely available for benchmarking and further research.
