# Supplementary material for: An Automated Literature Review Tool (LiteRev) for Streamlining and Accelerating Research Using Natural Language Processing and Machine Learning: Descriptive Performance Evaluation Study
Source: J Med Internet Res. 2023 Sep 15;25:e39736. doi: 10.2196/39736 (PMC10541641; doi:10.2196/39736)
Supplement: Multimedia Appendix 1 [file jmir_v25i1e39736_app1.docx]

Multimedia Appendix 1

Query for Embase:

('acute hiv':ab,ti OR 'early hiv':ab,ti OR 'primary hiv':ab,ti OR ('window period':ab,ti AND 'human immunodeficiency virus':ab,ti)) AND ('africa south of the sahara'/exp OR 'sub-saharan africa':ab,ti OR 'subsaharan africa':ab,ti OR 'africa south of the sahara':ab,ti OR angola:ab,ti OR benin:ab,ti OR botswana:ab,ti OR 'burkina faso':ab,ti OR burundi:ab,ti OR cameroon:ab,ti OR 'cape verde':ab,ti OR 'central africa':ab,ti OR 'central african republic':ab,ti OR chad:ab,ti OR comoros:ab,ti OR congo:ab,ti OR 'cote divoire':ab,ti OR 'democratic republic congo':ab,ti OR djibouti:ab,ti OR 'equatorial guinea':ab,ti OR eritrea:ab,ti OR eswatini:ab,ti OR ethiopia:ab,ti OR gabon:ab,ti OR gambia:ab,ti OR ghana:ab,ti OR guinea:ab,ti OR 'guinea-bissau':ab,ti OR kenya:ab,ti OR lesotho:ab,ti OR liberia:ab,ti OR madagascar:ab,ti OR malawi:ab,ti OR mali:ab,ti OR mayotte:ab,ti OR mozambique:ab,ti OR namibia:ab,ti OR niger:ab,ti OR nigeria:ab,ti OR rwanda:ab,ti OR sahel:ab,ti OR 'sao tome and principe':ab,ti OR senegal:ab,ti OR 'sierra leone':ab,ti OR somalia:ab,ti OR 'south africa':ab,ti OR 'south sudan':ab,ti OR sudan:ab,ti OR tanzania:ab,ti OR togo:ab,ti OR uganda:ab,ti OR zambia:ab,ti OR zimbabwe:ab,ti)

Query for Web of Science:

TS=(("acute hiv" OR "early hiv" OR "primary hiv" OR ("window period" AND "human immunodeficiency virus")) AND ("africa south of the sahara"/exp OR "sub-saharan africa" OR "subsaharan africa" OR "africa south of the sahara" OR angola OR benin OR botswana OR"burkina faso" OR burundi OR cameroon OR "cape verde" OR "central africa" OR "central african republic" OR chad OR comoros OR congo OR "cote divoire" OR "democratic republic congo" OR djibouti OR "equatorial guinea" OR eritrea OR eswatini OR ethiopia OR gabon OR gambia OR ghana OR guinea OR "guinea-bissau" OR kenya OR lesotho OR liberia OR madagascar OR malawi OR mali OR mayotte OR mozambique OR namibia OR niger OR nigeria OR rwanda OR sahel OR "sao tome and principe" OR senegal OR "sierra leone" OR somalia OR "south africa" OR "south sudan" OR sudan OR tanzania OR togo OR uganda OR zambia OR zimbabwe))

## Hyperparameters searches:

## Table S1. Hyperparameters definition, range and values for each clustering.

| **Parameters** | **Description** | **Range** | **First clustering** |
| --- | --- | --- | --- |
| PaCMAP n dimensions | Determine the dimensionality of the reduced dimension space that the data will be embedded into | 2 - 400 | 310 |
|  |  |  |  |
| PaCMAP n neighbours | Controls how PaCMAP balances local versus global structure in the data | 2 - 400 | 18 |
|  |  |  |  |
| hdbscan min cluster size | Represents the minimum number of papers that takes a cluster | 2 - 400 | 30 |
|  |  |  |  |
| hdbscan min samples | Represents how conservative the clustering is. A large value increases the amount of points labelled as noise; therefore, clusters will be more separated from each other | 2 - 400 | 7 |
|  |  |  |  |

| Parameters | Second clustering | Third clustering | Fourth clustering |
| --- | --- | --- | --- |
| PaCMAP n dimensions | 87 | 2 | 62 |
|  |  |  |  |
| PaCMAP n neighbours | 8 | 14 | 12 |
|  |  |  |  |
| hdbscan min cluster size | 18 | 12 | 9 |
|  |  |  |  |
| hdbscan min samples | 2 | 8 | 5 |
|  |  |  |  |
